# Genomic imbalance in endometrial hyperplasia and cancer

**DOI:** 10.1186/1897-4287-13-S1-A16

**Published:** 2015-09-09

**Authors:** Michal Bednarek, Maria Constantinou, Lukasz Kepczynski, Agata Shiar Kassassir, Anna Sobczuk, Maria Wieszczycka, Jacek Suzin, Bogdan Kaluzewski

**Affiliations:** 1 Chair of Clinical and Laboratory Genetics, Department of Clinical Genetics, Medical University of Lodd, Lodz, Poland; 2 Department of Gynaecology and Menopausal Diseases, Polish Mother's Memorial Hospital – Research Institute in Lodz, Lodz, Poland; 3 The 1st Chair of Gynaecology and Obstetrics and Department of Surgical & Oncological Gynaecology, Medical University of Lodz, Lodz, Poland

## 

Endometrial cancer belongs to the most frequently diagnosed malignant neoplasms of female genital organs, and its incidence is steadily growing. For the timebeing, no chromosomal aberrations have been determined unequivocally, which would be specific for the particular stages of endometrial hyperplasia and neoplastic transformation development.

The goal of the undertaken studies was an identification of the earliest and specific genetic changes, which could be attributed to an increased risk of neoplastic transformation in a group of patients with endometrial hyperplasia plus the characteristics of genetic changes associated with the mature form of neoplasm.

The study involved forty-four (44) patients, including five (5) histopathologically unconfirmed hyperplasia, twenty-six (26) with histopathologically confirmed endometrial hyperplasia and thirteen (13) with diagnosed endometrial cancer.

The applied aCGH (array Comparative Genomic Hybridisation) method enabled selection of a few chromosomal regions which indicated a higher incidence of chromosomal rearrangements than in the control group. The study included also an evaluation of the frequency of mutations of the genes specific for neoplastic transformation development, the genes at chromosomal loci, which most frequently presented with genomic imbalance.

In cases without hyperplasia, changes were diagnosed, described as CNVs (Copy Number Variations), which occurred with varying prevalence in the genome of the population of healthy subjects. Significant genomic imbalance was identified in 26 (100%) patients with diagnosed hyperplasia and in 11 (84.6%) of the patients with diagnosed endometrial cancer. Also other, till now unreported changes were found, localised at characteristic regions of the genome.

